# Neutrophil Extracellular Traps: A New Player in Cancer Metastasis and Therapeutic Target

**DOI:** 10.1186/s13046-021-02013-6

**Published:** 2021-07-16

**Authors:** Dakai Yang, Jing Liu

**Affiliations:** 1 Liver Disease and Cancer Institute, School of Medicine, Jiangsu University, Zhenjiang, People’s Republic of China; 2 Key Laboratory of Medical Science and Laboratory Medicine, School of Medicine, Jiangsu University, Zhenjiang, People’s Republic of China; 3 Microbiology and Immunity Department, Shanghai University of Medicine & Health Sciences, Shanghai, People’s Republic of China; 4 Collaborative Innovation Center for Biomedicines, Shanghai University of Medicine & Health Sciences, Shanghai, People’s Republic of China

**Keywords:** Neutrophil Extracellular Traps, Cancer metastasis, NETs formation, Targeting NETs

## Abstract

Neutrophil Extracellular Traps (NETs) are neutrophil-derived extracellular scaffolds, which typically consist of fibrous decondensed chromatins decorated with histones and granule proteins. Initially discovered as a host defence mechanism of neutrophil against pathogens, they have also been implicated in the progression of sterile inflammation-associated diseases such as autoimmune disease, diabetes, and cancer. In this review, we highlight and discuss the more recent studies on the roles of NETs in cancer development, with a special focus on cancer metastasis. Moreover, we present the strategies for targeting NETs in pre-clinical models, but also the challenging questions that need to be answered in the field.

## Background

Metastasis, although infrequent [1], remains the leading cause of cancer-related mortality (90%) in patients [2].Over the past decades, various mechanisms of metastasis have been revealed, including cancer cell intrinsic factors that endow the cells with sufficient motility/invasiveness [3–5], and the supportive microenvironment within the primary tumor as well as the metastatic sites that favors cancer cell survival, outgrowth and escape from immunosurveillance [6–8], yet so far cancer metastasis can neither be prevented at the early stage in high-risk patients nor eradicated during overt metastasis, highlighting an unmet medical need to develop new treatment regimens that effectively block metastatic cascade locally and systemically.

The observations that neutrophils frequently accumulate in peripheral blood and resected metastatic cancer tissues of advanced cancer patients [9, 10] suggest a possible association between neutrophils and metastasis. Extensive studies have confirmed the causal link between elevated neutrophil counts and increased risk for metastasis as well as poor prognosis [11, 12]. Indeed, neutrophils have been found to promote pre-metastatic niche formation [13, 14], facilitate metastatic colonization [15, 16] and dampen T cell-mediated immunity in metastatic cancer [17, 18]. Various mechanisms of neutrophil-mediated metastatic cascades have been elicited and among these neutrophil extracellular traps (NETs), an extracellular web-like structure released by neutrophils, have been recently identified as a novel mechanism contributing to different steps of metastasis and immune escape, therefore, may represent as a promising target in cancer therapy.

Interestingly, NETs was first discovered in 2004 as a host defence mechanism of bacteria trapping and killing [19]. As the most abundant immune cell type in the blood, neutrophils, upon activation by cytokines or bacterial endotoxin, release granule proteins and chromatin that together form extracellular fibers. The composition of NETs proteins varies in response to the stimulus and even the patients with the same disease phenotype displayed heterogeneity in NETs composition [20]. But the core proteins shared irrespective of the stimuli include histones, neutrophil elastase (NE) and myeloperoxidase (MPO), which are termed as NET core signature [20] and analysed as NETs markers in current research. The DNA-protein complexes can not only sequester Gram-positive and Gram-negative bacteria, but can also effectively kill them by degrading bacterial virulence factors with neutrophil proteases [19]. In this respect, the primary role of NETs is advantageous, providing a rapid and disseminated means of controlling infection. On the other hand, it is to note that excessive NETs production induced by neutrophil dysregulation is counterproductive and have been implicated in many inflammatory disorders, including autoimmune diseases [21] , type 2 diabetes [22] , myocardial infarction [23] and cancer [24, 25], which further exacerbate inflammation and cause cell damage.

In this review, with a special focus on cancer metastasis, we discuss recent studies on cancer-associated NETs formation and their role in promoting metastasis. We also present the newly developed strategy in targeting NETs or interfering NETs-cancer cell interactions as a potential treatment option for advanced cancer therapy.

## The General Formation Mechanism of NETs

Initially discovered in neutrophils though, the formation of extracellular traps do not exclusively confine to neutrophils. Indeed, many types of leukocytes [26–28], have been shown to produce extracellular traps as a defence mechanism in response to harmful stimuli. Therefore, the formation of extracellular traps is presumably a biologically conserved process. The molecular mechanisms of NETs formation, though still incompletely understood, is so far the most studied in this field.

Two main mechanisms of NETs formation have been described, depending on the death or live fate of neutrophils, and are termed as suicidal [29] or vital NET formation [30], respectively. As the name suggests, the suicidal NETs formation involves cell death where chromatin and antimicrobial proteins are expelled by dying neutrophils along with the rupture of cytoplasmic membrane. However, distinct from apoptosis and necrosis, this unique form of neutrophil death resulting in NETs formation is insensitive to caspase inhibition and necrostatin [31], thus termed as NETosis according to Nomenclature Committee on Cell Death [32]. Notably, not all NETs formation requires the process of cell death. Indeed, neutrophils can also release NETs in a very rapid (within minutes) and cell death–independent manner, rendering intact anuclear neutrophils [33], suggesting a coordinated defence mechanism of neutrophils against invading bacteria (Table 1 and Fig. . 1).

**Table 1:** Types and Characteristics of NETs Formation

	NETosis		Viable NETs Formation	
	NOX2-Dependent	NOX2-Independent	ROS-Dependent	ROS-Independent
Stimulus	PMA; LPS	Calcium or Potassium ionophore; Nicotine	LPS; C5a	Staphylococcus aureus
Intermediate Signaling	PKC-Raf-Mek-Erk	SK channel	ND	ND
Dependent on	NOX2-ROS, MPO, NE	MitoROS, Akt, PAD4	ROS	ND
Independent of	PAD4	NOX2, MPO, NE	ND	ROS
DNA Content	Decondensed chromatin	Decondensed chromatin or mtDNA	mtDNA	Decondensed chromatin
Time Period for NETs Formation	2.5–4 h	3–4 h	15 min	5 min
Neutrophil Destiny	Lysis	Lysis	Alive, Loss of mtDNA	Alive, Anuclear

*NOX2* NADPH oxidase 2, *PMA* phorbol 12-myristate 13-acetate, *ROS* reactive oxygen species, *LPS* lipopolysaccharide, *ERK* extracellular-signal-regulated kinase, *MEK* MAPK/ERK kinase, *mitoROS* mitochondrial reactive oxygen species, *MPO* myeloperoxidase, *NE* neutrophil elastase, *PAD4* protein-arginine deiminase type 4, *PKC* protein kinase C, *SK channel* small conductance calcium-activated potassium channel, *TLR* Toll-like receptor, *ND* not determined

**Fig. 1:**
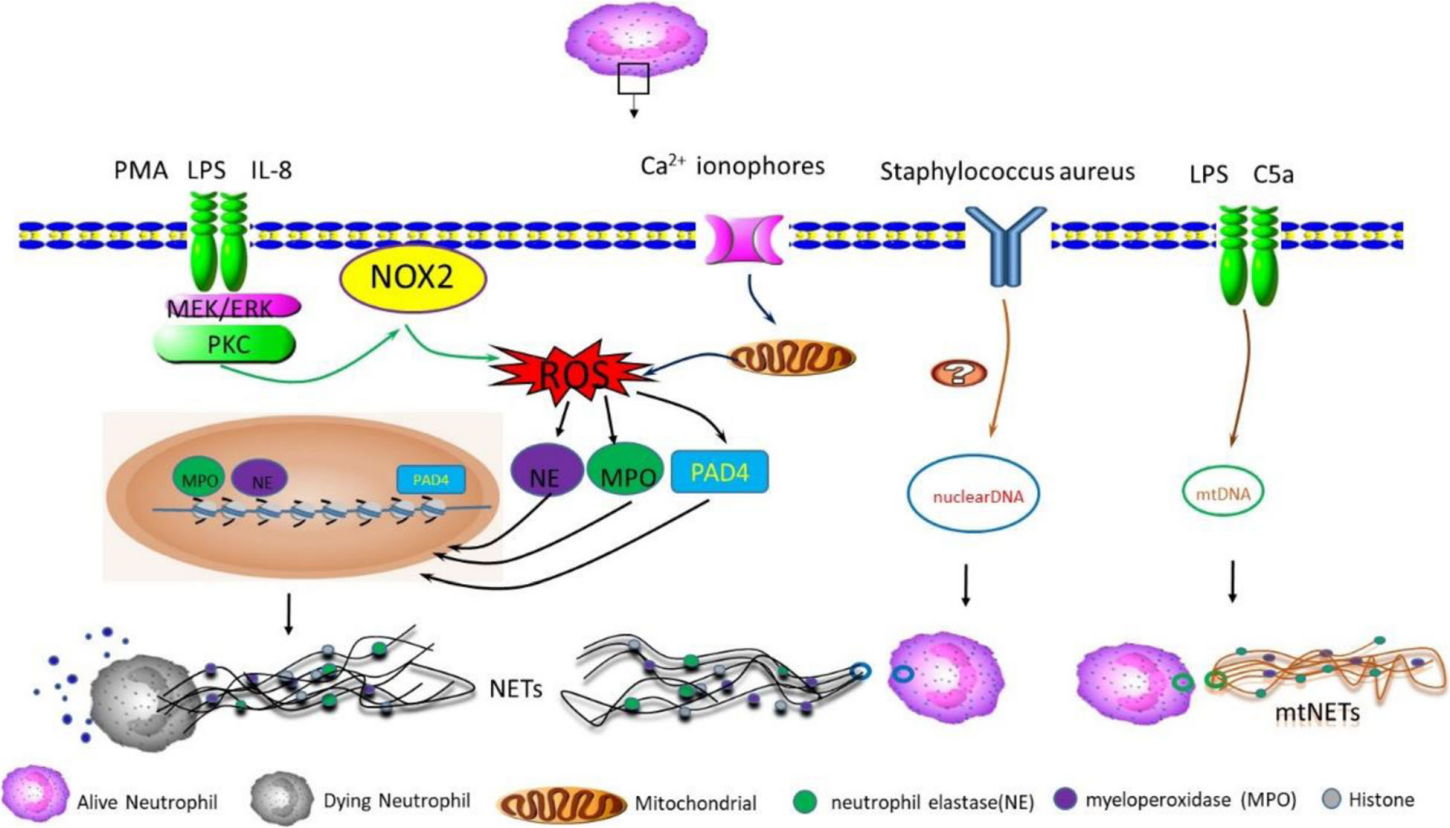
**NETs formation pathways**. Generally speaking, Neutrophil extracellular traps (NETs) form via two pathways depending on the stimuli. The first involves neutrophil death termed as NETosis. Activated neutrophils in response to PMA, LPS and IL-8 induces NADPH Oxidase (Nox2)-mediated production of reactive oxygen species (ROS). ROS then triggers the downstream signalling cascade for chromatin decondensation, including neutrophil elastase (NE) and myeloperoxidase (MPO), which translocate into the nucleus and cooperate for chromatin decondensation. ROS also activates protein-arginine deiminase type 4 (PAD4), which hypercitrullinates histones, contributes to chromatin decondensation. Decondensed chromatin are then released into the cytoplasm, mixing with granule proteins and forming NETs. Finally, the plasma membrane permeabilizes, allowing for NETs expulsion while the neutrophil dies. The second pathway is non-lytic NETs formation that occurs in response to calcium ionophores or Staphylococcus aureus in the absence of cell lysis. Similar to the budding of extracellular vesicles, this process involves the release of nuclear DNA or mitochondrial DNA (mtDNA) bound vesicles into the extracellular space where they are ruptured, leaving the neutrophil alive without nuclear or loss of mtDNA

### NADPH Oxidase-Dependent suicidal NETs Formation

As the first discovered and classical mechanism of NETs formation, the NADPH oxidase-dependent suicidal NETs formation has been reported to occur primarily at 3 to 8 hours following the activation of neutrophils by various stimuli, such as phorbol 12-myristate 13-acetate (PMA), lipopolysaccharide (LPS) and interleukin-8 (IL-8) [34]. Within this period, neutrophils undergo a series of biochemical processes, including nuclear delobulation, disassembly of the nuclear envelope, chromatin decondensation and eventually disintegration of plasma membrane, leaving the neutrophil to die, while the chromatin and granulated proteins to expulse [29].

Chromatin decondensation is a key step during NETs formation. The production of reactive oxygen species (ROS) mediated by NADPH Oxidase 2 (Nox2) have critical roles in NETosis as it triggers the downstream signalling cascade for chromatin decondensation. MPO and NE, two key enzymes stored in azurophilic granules of naïve neutrophils, are stimulated by ROS generation [35]. Activated NE then translocates into the nucleus and disrupt chromatin packaging by degradation of core histones. Subsequently, MPO binds to chromatin and cooperate with NE for further decondensation [35]. In addition, protein-arginine deiminase type 4 (PAD4), which hypercitrullinates histones, is implicated to be another downstream effector of ROS and contributes to chromatin decondensation [36]. Decondensed chromatin are then released into the cytoplasm, mixing with granule proteins and forming NETs. Finally, the plasma membrane permeabilizes, allowing for NETs expulsion while the neutrophil dies.

### NADPH Oxidase-independent suicidal NET Formation

Intriguingly, NETs formation has also been observed in patients with chronic granulomatous disease [29], an inherited disorder in which NADPH oxidase activity is lacking and results in defective generation of ROS, raising the question of ROS necessity in NETs formation. In fact, NADPH Oxidase-independent NETosis also exists, but the ROS generation is alternatively provided by mitochondria rather than NADPH Oxidase. Some stimuli, such as immune complexes [37], calcium ionophores [38] and nicotine [39], have been implicated to trigger NETosis via mitochondrial ROS (referred as mitoNETs). More importantly, mitoNETs, in some autoimmune diseases, are enriched in oxidized mitochondrial DNA instead of nuclear chromatin which acts as potent inflammatory inducer and suggests a novel mechanism of neutrophil-mediated inflammation in autoimmune diseases [37].

### Viable NETs formation

Non-lytic NETs formation, still poorly understood, on the other hand, is a faster neutrophil response without loss of nuclear or plasma membrane, and can be ROS-dependent or independent. Gram-positive bacteria such as Staphylococcus aureus, has been found to trigger viable NETs formation independent of oxidant, generating NETs DNA primarily of nuclear origin [33]. Similar to the budding of extracellular vesicles, this process involves the release of nuclear DNA bound vesicles into the extracellular space where they are ruptured and the chromatin is released [33]. Another type of vital NETs formation resulting in mitochondrial DNA-containing NETs have also been observed in vitro when neutrophils are stimulated with LPS or complement factor 5a (C5a) [30]. Since previous study also reports a suicidal NETs formation with LPS treatment, the vital NETs formation induced by LPS may suggest a complementary mode of NETs formation at different stages in response to stimuli, which coordinately contribute to host defence against invaders.

## NETs formation in the context of cancer

Normally induced by pro-inflammatory factors during infection, NETs can also be stimulated by cancer cells and cancer-associated fibroblasts (CAF) in the absence of infection [40] , which hijacks antimicrobial immune system for cancer cell proliferation and metastasis (Table 2). The crosstalk between tumor cells and neutrophils can be mediated by tumor-derived chemokines, including granulocyte colony-stimulating factor (G-CSF) [41, 42], IL-8 [43, 44] and CXC Chemokine Receptor (CXC) ligands [45], which have been implicated in protumour NETosis in multiple murine models. In addition to chemokines, tumoral Cathepsin C, a cysteine protease essential for catalytic activation of serine proteases, has been shown to induce neutrophil ROS production and IL-1β secretion, thus promoting neutrophil infiltration and NETs formation [46] . Not only cancer cell-derived factors can drive NETosis locally or systemically, but also cancer-associated fibroblasts (CAF) have recently been identified as a key mediator in suicidal NETosis. Munir et al found that CAF-derived Amyloid β was sufficient to induce local or systemic NETosis via its proposed cognate receptor CD11b on neutrophils, which in turn reciprocally activate CAFs with enhanced proliferation and contraction ability [47]. Interestingly, unlike previous reports which showed a central role of tumor-derived G-CSF in priming neutrophils towards NETs generation, CAF-mediated NETosis in this study does not require G-CSF, suggesting a possible distinct mechanism involved in the stromal cell-induced NETs formation. Considering neutrophils can be primed under various stimuli, such as hypoxia [48], autophagy [49], surgical stress [50] or inflammation [51], which also manifests the hallmarks of cancer development, it would be interesting to investigate tissue and stage-specific mechanisms of NETs formation during cancer progression.

**Table 2:** Mechanisms of NETs Formation in the Context of Cancer

Mouse model	Detected NETs marker	NETs Formation Mechanism	Reference
Lewis lung carcinoma; Breast carcinoma; CML	WB/IF: H3Cit; IF: DNA Plasma DNA Count; FACS: H3Cit^+^ Neutrophils	Cancer cell-derived G-CSF	[41]
Melonoma; Lewis lung carcinoma	WB/IF: H3Cit; IF: DNA	Cancer cell-derived G-CSF	[42]
Colorectal cancer liver metastasis	IF: MPO^+^ /H3Cit+/extracellular DNA^+^	Tumor-derived IL-8	[43]
Gall bladder cancer	IF: NE^+^ /H3Cit+/extracellular DNA^+^	Cancer cell-derived IL-8	[44]
Lewis lung carcinoma; Breast carcinoma; Colon carcinoma	IF: H3Cit+/extracellular DNA^+^	Cancer cell-derived CXCR1 and CXCR2 ligands	[45]
Lung metastasis of breast cancer	IF: MPO^+^ /H3Cit+/extracellular DNA^+^	Cancer cell-derived Cathepsin C	[46]
melanoma Pancreatic adenocarcinoma Lung adenocarcinoma	IF: MPO^+^ /H3Cit+/extracellular DNA^+^	Stroma-derived Amyloid β	[47]

*WB* Western Blot, *IF* Immunofluorescence, *H3Cit* citrullinated histone H3, *MPO* myeloperoxidase, *NE* neutrophil elastase, *CXCR* CXC Chemokine Receptor

## NETs promote metastasis

**Fig. 2:**
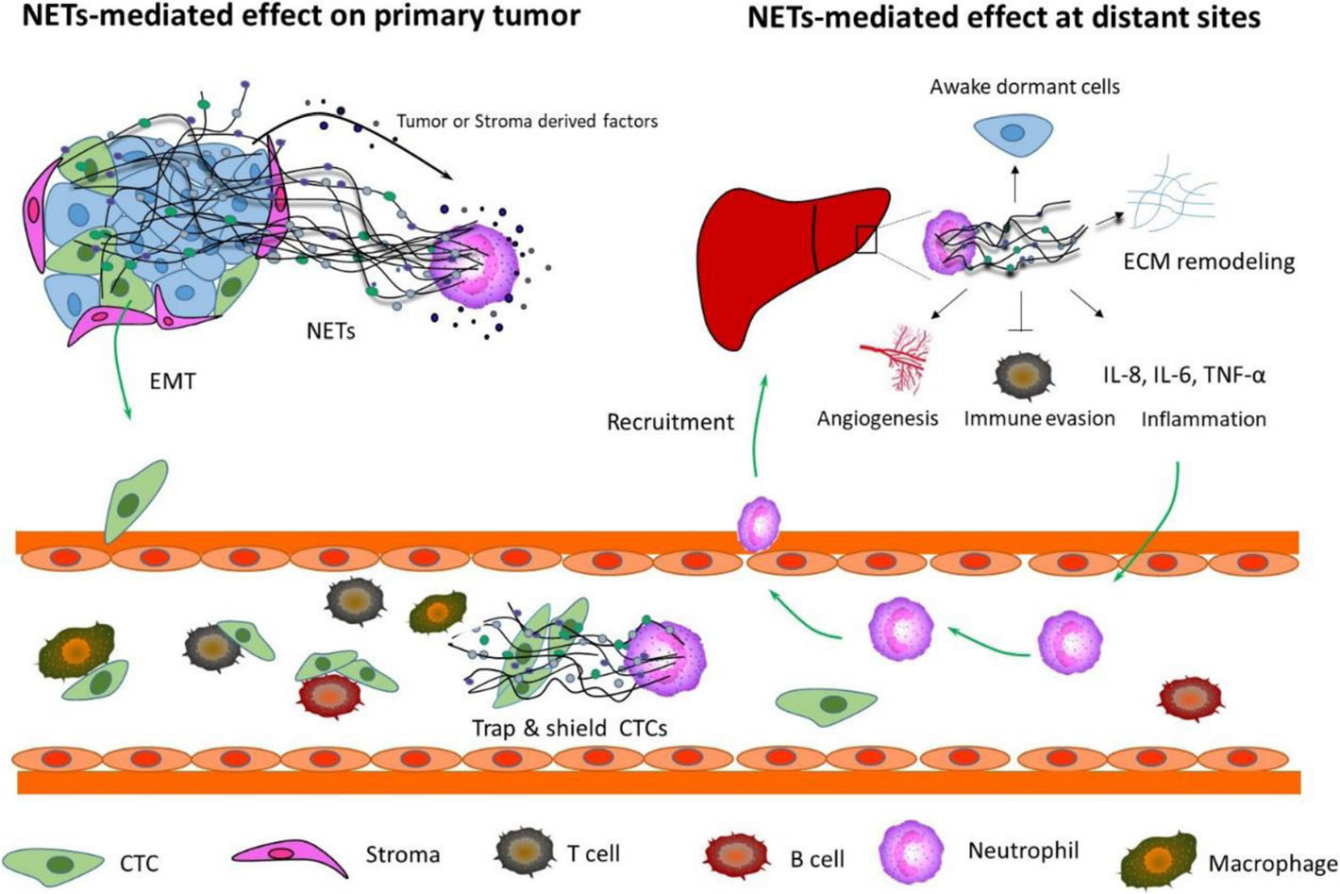
**NETs promote metastasis both on primary tumor and at distant sites.** NETs can induce the Epithelial-Mesenchymal Transition (EMT) of primary tumor cells, which arms the cancer cells with motility and invasiveness. The cancer cells invading into the circulation are termed as circulating tumor cells (CTCs), which are shielded and protected by NETs from the attack of various immune cells and fluid shear forces. NETs can also modulate the pre-metastatic niche, including the establishment of an inflammatory and immune evasion milieu, the enhancement of Angiogenesis and vascular permeability. In addition, NETs can drive the dormant cancer cells re-entering the cell cycle and thus reinitiation of aggressive metastatic outgrowth

NETs have been shown to participate in the dissemination of cancer cells, the initial steps of the metastatic cascade that arms neoplastic cells with the ability to invade the local microenvironment, survive in circulation and seed in secondary organs. Moreover, during the journey to distant organs, cancer cells encounter various obstacles including the endothelial barrier, immune surveillance, and metabolic/nutrient stress before a successful metastatic colonization [1]. Emerging evidence have described how NETs facilitate the different steps of the metastatic cascade (Fig. 2).

### The Epithelial-Mesenchymal Transition

One centrally important process arming the cancer cells with motility and invasiveness is the epithelial-mesenchymal transition (EMT). The ability of NETs to induce EMT is first described in vitro when the coincubation of normal endothelial cells with NETs isolated from human neutrophils stimulated with PMA leads to the loss of endothelial cell junction, the development of mesenchymal phenotype and aberrant activation of the Wnt/β-catenin signaling pathway [52]. Following this study, NETs-mediated EMT induction has been replicated in neoplastic epithelial cells as well as in murine models [53, 54], resulting in an even more significant exhibition of invasive phenotypes with high expression of EMT master regulators, including Snail, Slug and Zeb, when compared with known EMT stimulants such as TGF-β and IL-8 [55]. The ability of NETs to drive EMT both in fully normal epithelial cells and in neoplastic epithelial cells suggests that NETs may serve as one of the early contributors during the process of neoplastic transformation. More importantly, the correlation of NETs deposition with EMT activation has been confirmed in primary tumor as well as metastatic biopsy, though the mechanism of NETs-driven EMT is still not clear.

Intriguingly, Martins-Cardoso et al found that DNase treatment resulting in the destruction of NETs integrity exhibited minor impact on the invasive and migratory ability of breast cancer cells, suggesting a dispensable role of NETs integrity on EMT induction [53]. Consistently, Kajioka et al also found that degradation of NETs DNA does not affect the migration and invasion of the primary tumor whereas pharmacological inhibition of NET-associated elastase or NET-derived HMGB1 abrogates EMT induction [54], proposing the NETs proteins as the main contributor to EMT induction. Notably, degradation of NETs-DNA with DNase, though showed little influence on EMT, have been found to attenuate metastasis [54], suggesting NETs contribution to other steps of metastatic cascade, in addition to EMT.

### Cancer Cell adhesion

Neoplastic cells derived from the primary site acquire the migration ability through EMT and may, sooner or later, invade into the circulation where they are termed as circulating tumor cells (CTCs) [1]. Though a surprisingly high amount of CTCs has been found in patients’ circulation, only a minority survive and form macrometastases under exposure of fluid shear forces, innate immunity and oxidative stress [1]. Therefore, early entrapment of CTCs could be one of the protective mechanism rendering their arrest in the circulation during transit. In accordance with the role of NETs in trapping microorganisms, researchers found that microvascular NETs deposition in response to cancer-associated inflammation is able to sequester CTCs. However, different from their trapping and killing pathogen, NETs-trapped cancer cells actually displayed enhanced invasiveness and subsequent metastasis potential [56] . Further investigation found that trapped cancer cells could internalize NETs via toll like receptor TLR4/9, leading to the activation of inflammatory signalling such as NFκB [56], MAPK/p38 and STAT3 pathway in cancer cells [57] and thereby facilitates metastatic outgrowth. In addition to the uptake of NETs via classical DAMP sensors on cancer cell, various mechanisms have been proposed, including the mechanical entrapment with web-like structure of NETs DNA [58], the integrin-mediated tethering [59] and the adhesion molecule-driven retention of CTCs [60]. Moreover, the accumulation of NETs in distant organs may serve as a directional chemotaxis factor for the recruitment of CTCs, as observed in a recent study where NETs DNA abundant in liver metastasis of breast cancer and colon cancer patients, acts as a chemotactic factor to attract CTCs through coiled-coil domain containing protein 25 (CCDC25), a transmembrane protein present on cancer cells [25], providing a possible mechanism to explain the site preference of metastasis and a therapeutic target in inhibiting metastasis .

### NETs modulate pre-metastatic niche

After survival in the circulation and infiltration into the distant organs, newly disseminated tumor cells (DTCs) are immunologically and metabolically vulnerable to the challenging microenvironment in distant organs. The seed and soil hypothesis provides an explanation for organotropism in metastasis [8]. Indeed, the permissive and supportive pre-metastatic niche (the “soil”) are cultivated prior to the arrival of DTCs (the “seed”), thus rendering distant organs hospitable for tumor cell extravasation, proliferation, and colonize [3]. The major players involved in the initiation and formation of pre-metastatic niche includes primary tumor cells, bone marrow-derived cells and host stromal cells. In particular, emerging evidence demonstrated the recruitment of neutrophils into pre-metastatic niche such as liver [25], lung [61] or omentum [14], provides a permissive niche for the incoming cancer cells, at least in part through NETs-mediated remodelling of the local microenvironment.

#### Inflammation

One of the key characteristics of pre-metastatic niche is inflammation. Evidence for a role of NETs in the establishment of an inflammatory milieu at secondary sites is emerging, which promotes seeding, survival, and proliferation of primary tumor cells to the metastatic sites. For example, a recent finding observed a positive correlation of NETs level with colorectal cancer metastasis. Further studies showed that neutrophil recruitment in response to primary tumor-derived factors results in the over-production of NETs in the liver, which is able to capture colorectal cancer cells (CRC) and subsequently induce the production of pro-inflammatory cytokines such as IL-8, IL-6 and TNF-α in trapped CRC [43]. The inflammatory microenvironment in turn recruits more neutrophils with NETs deposition, thus forming a vicious cycle connecting NETs and inflammatory niche in CRC liver metastasis.

#### Tumor immune Evasion

Local immune defence against infiltrating cancer cells are mainly executed by cytotoxic CD8 ^+^ T cells and NK cells, however, that function is commonly impaired in patients with advanced cancer. Recent study uncovered a NET-mediated physical barrier in immune evasion where NETs wrap and shield tumor cells from interactions with neighboring anti-tumor immune cells, thus impeding the activity of CD8^+^ T cells and NK cells against tumor [45, 62].

#### Angiogenesis and vascular permeability

The pre-metastatic niche may enhance angiogenesis and vascular permeability to facilitate metastatic colonization. Though the direct evidence of NETs in angiogenesis and vascular permeability in the context of tumor is scarce, a functional link between NETs and angiogenesis has been implicated in tissue repair [63] as well as in vascular pathology, including pulmonary hypertension [64] and corneal neovascularization [65]. The possible mechanism could be NETs-mediated activation of endothelial cells via TLR-4/ NFκB signalling [64], inflammation-induced upregulation of proangiogenic factors such as vascular endothelial growth factor (VEGF) [65, 66] , and the newly identified mechanism relying on the NETs clearance of senescent endothelial cells [67]. In addition to angiogenesis, NETs have been found to increase vascular permeability by disrupting the endothelial barrier. NETs-associated proteases, including NE, MPO and matrix metalloproteinases (MMP), compromise junction integrity via the cleavage of vascular endothelial (VE)-cadherin [68] and glycocalyx [69] and thus promote vascular permeability.

### NETs awake the dormant cancer cells

Intriguingly, most DTCs that have managed to extravasate and disseminate to distant organs are destined to enter into a state of dormancy, sometimes for weeks, months, even years or decades, before forming metastasis [3]. Dormancy program confers a growth-arrested state in single DTCs or in small micrometastatic clusters, which actually reflects plasticity of DTCs to adapt to a given tissue and results in immune evasion and radiological undetectable [3]. Unfortunately, dormant cells will eventually exit the quiescent state and produce overt metastasis, raising the fundamental question of what signalling pathways or cellular events terminate the dormancy program. Albrengues [24] *et al* unveiled a NETs-initiated reactivation of dormant tumor cells in multiple metastasis models. Mechanistically, this effect is not via the direct contact of NETs and tumor cells. Instead, remodelling of extracellular matrix (ECM) by NETs-associated NE and MMP9 are mainly responsible for the proliferation of DTC to form metastasis, whereby the cleavage of laminin generates a new epitope which could be sensed by the integrin present on dormant cancer cells. The integrin-mediated interaction of ECM and dormant cancer cells subsequently activates the downstream effectors such as focal adhesion kinase (FAK), mitogen-activated extracellular signal-regulated kinase (MEK) and extracellular signal regulated kinase (ERK), thus leading to the dormant cancer cells re-entering the cell cycle and reinitiation of aggressive metastatic outgrowth [24].

## Therapeutic implications

**Fig. 3:**
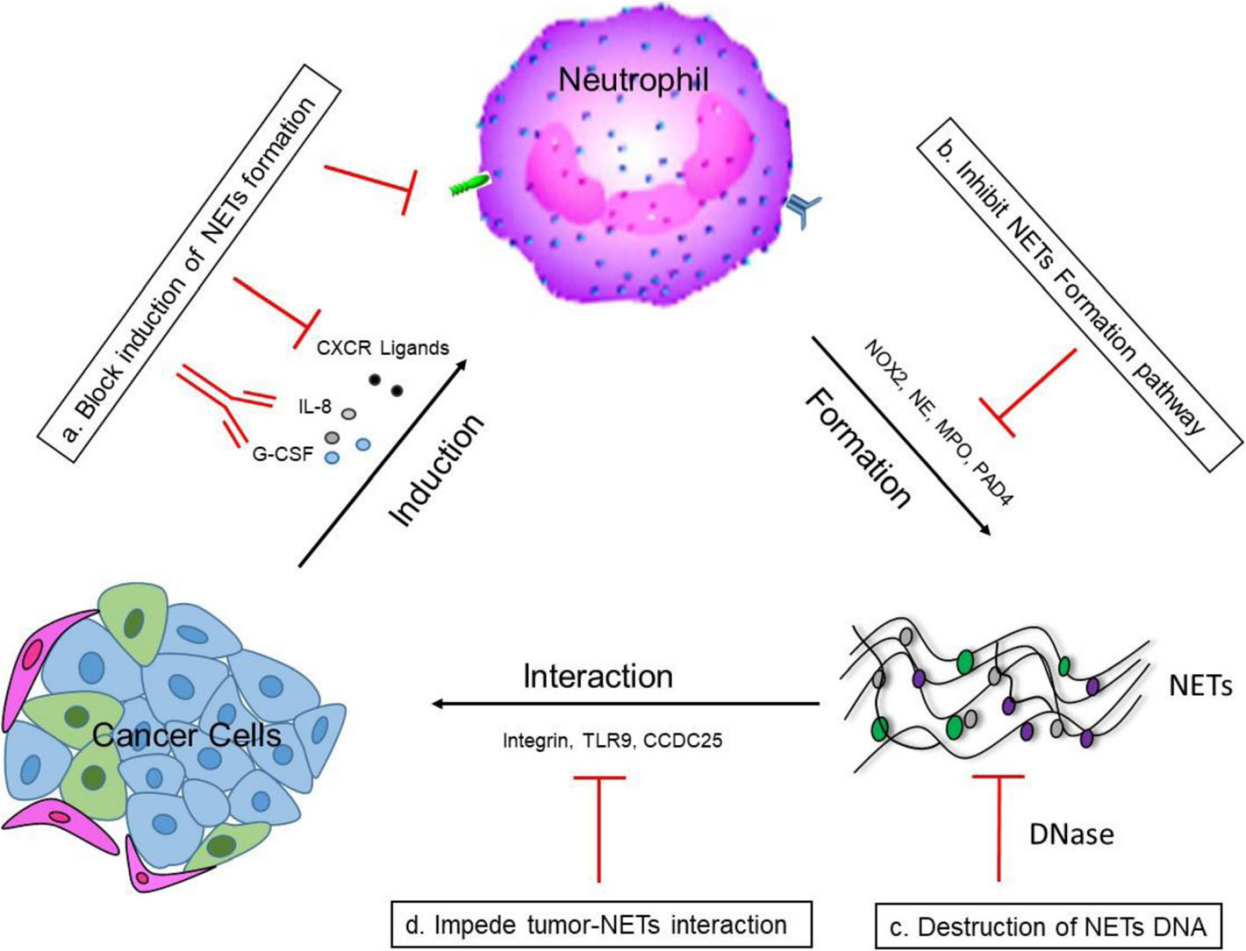
The strategy of targeting NETs or blocking NETs-mediated effect. **a** The induction of cancer-associated NETs formation is mediated by the interaction of cancer or stroma –derived chemokines/cytokines and the specific receptor present on neutrophils. Blockade of the chemokines/cytokines or the receptors on neutrophils can inhibit the induction of NETs formation. **b** Inhibition of NETs formation via targeting key players such as NOX2, NE, MPO or PAD4 in the formation pathway. **c** Direct destruction of NETs structure via DNase. **d** NETs-mediated pro-tumor effect is through the interaction of NETs and cancer cells. Block their interaction via targeting the mediators can prevent NETs effect on cancer cells

The ability of NETs to foster metastatic dissemination and immune evasion highlights the potential for NETs-targeted therapies to inhibit metastasis and boost efficacy of immunotherapy. Targeting tumor-associated neutrophils can be desirable, however, the risk of severe and even fatal infections found in patients caused by neutrophil depletion limits its clinical use [70]. In this sense, NETs represents a more promising therapeutic target. Currently, therapeutic approach to block NETs are mainly through the direct disruption of NETs structure or interference with the pathway of NETs formation. Moreover, with the mechanistic illustration of the interplay between cancer cell and NETs, specific blockade of their interaction have shed light on a new strategy to curb NETs-mediated metastasis [25] (Fig. 3).

### Inhibition of general NETs formation pathway

To study the effects of NETs deficiency on tumor metastasis, conventional and neutrophil-specific PAD4 knockout mice have been generated and exhibited defects in NETosis. While neutrophil infiltration was not affected by the NETs depletion, metastatic colonization was dramatically reduced, compared with NETs-competent mice [14, 61, 71, 72]. Furthermore, several PAD4 inhibitors, including the specific inhibitor (GSK484) and the pan-pad inhibitor (Cl-amidine) have been tested in preclinical studies [14, 45] and displayed similar anti-metastatic effect as shown in PAD4-deficient mice. Given the critical role of NE and MPO in NETosis and as core protein components in NETs structure, NE-deficient mice or NE inhibitors have been utilized to study the effects of NETs depletion on metastasis. In a sepsis-associated lung carcinoma model, micrometastases was abrogated by the treatment of NE inhibitor GW311616A with markedly decreased tumor cell adhesion to hepatic and pulmonary microvasculature [58]. However, it is to note that the short serum half-life of the PAD4 and NE inhibitors currently available greatly limits their application in clinic and the development of new inhibitors with extended half-life is required. In addition, it is found that mice lacking NADPH oxidase [73] or treated with MPO inhibitor [74] fail to form NETs, which may also be utilized as a preclinical model to evaluate the effects of NETs absence in metastasis.

### Targeting tumor-associated Induction of NETs formation

The context-specific pathway of NETs induction provides attractive target for interference of NETs formation under different malignancy, as exemplified by blocking IL-8 [43], Cathepsin C (CTSC) [46], Amyloid β [47] or CXCR-1, -2 [45], which are either tumor-derived pro-inflammatory factors or the receptors on neutrophils responsible for NETosis upon activation. Preventing neutrophils from receiving tumor-associated NETs stimuli can not only prevent metastatic colonization, but also synergize immunotherapy by destroying the shielding NETs of tumor cells. Importantly, compared with targeting PAD4 or NE, this strategy would specifically impede pathological NETosis without affecting other critical neutrophil functions. Therefore, a comprehensive understanding of the tumor-associated induction of NETs formation is a prerequisite for specifically inhibition of NETs generation.

### Destruction of NETs DNA

Destruction of NETs structure at the primary or metastatic sites may represent another effective approach, which have the advantage of preserving antimicrobial functions of NETs in non-tumorous tissues. DNA, the backbone of the NETs structure, is therefore a feasible target for NETs degradation. Indeed, DNase I treatment is approved by FDA for the treatment of cystic fibrosis, partly attributed to the reduction of mucus viscosity caused by excessive NETs deposition during chronic inflammation [75]. Previous studies in cancer research have confirmed the potent anti-metastatic effect in preclinical models exerted by administration of recombinant DNase I [72, 76, 77], which however, requires a long-term multiple treatment due to the short half-life of DNase I in circulation [76, 77].

Recent progress have been made by crosslinking DNase I with nanoparticles to extend its half-life. As expected, treatment of DNase I-coated nanoparticles showed higher blood concentration, compared with free DNase I, but a continuous injection of the nanoparticles for two weeks is still required for the reduction of the lung metastatic burden [40]. Therefore, gene therapy may provide a feasible alternative approach to DNase I protein treatment. More importantly, the modification of gene vectors is able to achieve tissue-specific expression of transgene. Xia et al has developed a liver-specific DNase I delivery system using hepatotropic adeno-associated virus (AAV) vectors. A single administration of AAV-DNase I achieved a sustained liver expression of DNase I, which effectively inhibit CRC liver metastases along with enhanced antitumor immune response [78].

### Block NETs-cancer cell interactions

A growing body of evidence has suggested that NETs act more than a physical shield to cancer cells. Indeed the crosstalk of NETs and cancer cells promotes the invasiveness and migration ability through specific signalling. Therefore, some groups have focused on the interaction mediators present on NETs, cancer cells or both and explored the effects of inhibiting their interactions on metastasis. To date, the discovered mediator includes adhesion molecules such as integrin [79] and carcinoembryonic Ag cell adhesion molecule 1(CEACAM) [60], toll like receptor 9 [57], and CCDC25 [25]. Functional blocking these mediators using neutralizing antibody, specific antagonist, or the gene-deficient mice have exhibited reduced tumor cell migration and adhesion to the pre-metastatic niche even in the presence of widespread NETs deposition. Still it is to note that inhibition of one mediator in some cases may not completely prevent the NETs-tumor cell interaction as other proteins expressed on NETs or tumor cell can also contribute to their interactions [79], which makes this approach more complicated than direct destruction of NETs.

## Conclusions

The contribution of NETs to metastatic process has gained a growing appreciation and the approaches to targeting NETs deposition exhibited beneficial effects both in primary and metastatic tumors, which, however, has been challenged by a recent finding demonstrating an opposite effect of NETs to suppress tumor growth via the activation of immune response against tumor [80]. This seeming discrepancy reflects we are in the early stage of NETs study facing fundamental questions and a better understanding of the underlying mechanism is urgently needed. The major one is linked with the phenotypic diversity of neutrophils. Cancer-related neutrophils (both circulating and tumour-associated) can be primed towards antitumor (N1) or protumor (N2) phenotype under different cues, raising an important question of whether and how the functional plasticity of neutrophils predispose the released NETs with protumor or antitumor traits. If the function of NETs is determined by the neutrophil phenotype, it may help to explain the seeming paradox that Bacillus Calmette-Guerin (BCG)-induced NETs formation prevent tumor growth and migration while NETs induced by G-CSF promote tumor growth since BCG vaccination has been shown to induce trained immunity in neutrophils [81] whereas G-CSF was shown to direct neutrophil polarization toward the N2 phenotype [82].

Second, if there exists some association of neutrophil phenotypes with NETs functions, it will give rise to another puzzling that how the divergent functions of NETs is executed since the DNA backbone and core proteins are found to be the main mediator of NETs biological roles, according to current research. The possible mechanism could be related to some non-core proteins within NETs which has been overlooked. Indeed, these non-core proteins can not only define the uniqueness of NETs in terms of neutrophil sources, stimuli as well as action sites, such as NETs deposited in vessels or tissues, but may also determines their functional specificity. Thus NETs proteomes would be very helpful for the comprehensive understanding of the proteomic profiles of NETs. Another perspective is attributed to a dose-dependent effect, as aberrant NETs deposition in chronic inflammation often leads to the detrimental role. Additionally, it has been found that NETs stability and integrity varies under different stimuli, which may also results in their functional differences.

Finally, some limitations exists in the current study setting. For example, NETs are mostly isolated from neutrophils of healthy volunteers in response to PMA stimulation, which may not represent the NETs released in vivo under multitude stimuli in cancer patients. Besides, current studies on metastasis mainly rely on xenograft or orthotopicaly transplanted immune-compromised mouse models, which cannot recapitulate the complex relationship between the tumor and the microenvironment, therefore, immune-competent mouse model or humanized mouse model, if available, would be more desirable to study the effects of NETs on metastasis.

## Abbreviations

NETs: Neutrophil extracellular traps
NE: Neutrophil elastase
MPO: Myeloperoxidase
PMA: Phorbol 12-myristate 13-acetate
LPS: Lipopolysaccharide
IL-8: Interleukin-8
ROS: Reactive oxygen species
Nox2: NADPH Oxidase 2
PAD4: Protein-arginine deiminase type 4
C5a: Complement factor 5a
G-CSF: Granulocyte colony-stimulating factor
CXC: CXC Chemokine Receptor
EMT: Epithelial-mesenchymal transition
CTCs: Circulating tumor cells
CCDC25: Coiled-coil domain containing protein 25
DTCs: Disseminated tumor cells
CRC: Colorectal cancer cells
MMP: Matrix metalloproteinases
VEGF: Vascular endothelial growth factor
ECM: Extracellular matrix
FAK: Focal adhesion kinase
ERK: Extracellular signal regulated kinase
MEK: Mitogen-activated extracellular signal-regulated kinase
CEACAM: Carcinoembryonic Ag cell adhesion molecule
AAV: Adeno-associated virus
BCG: Bacillus Calmette-Guerin
